# Determination of per- and polyfluoroalkyl substances (PFAS) in six different fish species from Swiss lakes

**DOI:** 10.1007/s00216-024-05524-1

**Published:** 2024-10-01

**Authors:** Mylène Soudani, Lucie Hegg, Camille Rime, Camille Coquoz, Denise Bussien Grosjean, Francesco Danza, Nicola Solcà, Fiorella Lucarini, Davide Staedler

**Affiliations:** 1 TIBIO Suisse Romande, Chemin de Bérée 4C, 1010 Lausanne, Switzerland; 2 Center for Primary Care and Public Health (Unisanté), University of Lausanne, Route de La Corniche 2, 1066 Epalinges-Lausanne, Switzerland; 3 TIBIOLab Sàrl, Route d’Yverdon 34, 1373 Chavornay, Switzerland; 4 Département de La Mobilité, du Territoire Et de L’environnement (DMTE), Service de L’environnement (SEN), Avenue de La Gare 25, 1950 Sion, Switzerland; 5 Direction de L’environnement Industriel, Urbain Et Rural, Direction Générale de L’environnement, Etat de Vaud, Chemin Des Boveresses 155, 1066 Epalinges, Switzerland; 6 Dipartimento del Territorio, Divisione Dell’Ambiente, Sezione Della Protezione Dell’aria Dell’acqua E del Suolo, Via Franco Zorzi 13, 6501 Bellinzona, Switzerland; 7 School of Engineering and Architecture of Fribourg, Institute of Chemical Technology, HES-SO University of Applied Sciences and Arts of Western Switzerland, Boulevard de Pérolles 80, 1700 Fribourg, Switzerland; 8 Department of Biomedical Sciences, University of Lausanne, Rue du Bugnon 27, 1011 Lausanne, Switzerland

**Keywords:** Perfluoroalkyl and polyfluoroalkyl substances, PFAS, Fish fillet, QuEChERS, LC–MS/MS, Environmental pollutants

## Abstract

Per- and polyfluoroalkyl substances (PFAS) are persistent environmental contaminants with bioaccumulation potential, particularly affecting aquatic ecosystems and human health also via fish consumption. There is therefore a need for reliable extraction methods and studies to accurately assess PFAS levels in fish, crucial for understanding bioaccumulation and potential toxicological effects on both fish and humans through consumption. This study investigated PFAS levels in freshwater fish from Swiss lakes, focusing on six common species: *Coregonus wartmanni*, *Cyprinus carpio*, *Oncorhynchus mykiss*, *Perca fluviatilis*, *Salmo trutta*, and *Squalius cephalus*. Utilizing an optimized QuEChERS extraction method, 15 PFAS were analyzed in 218 fish fillet samples using liquid chromatography-mass spectrometry (LC–MS/MS). The results were compared to EU regulations and EFSA guidelines for tolerable weekly intake (TWI), with a specific focus on correlations between fish size and PFAS concentration. Our findings reveal significant PFAS contamination, particularly in *Perca fluviatilis* with perfluorooctane sulfonic acid (PFOS) and perfluorohexane sulfonic acid (PFHxS) levels often exceeding EU safety limits. TWI, calculated for a person of 70 kg body weight and an intake of 200 g of fish fillet, is exceeded in 95% of *Coregonus wartmanni*, 100% of *Squalius cephalus*, and in 55%, 50%, and 36% of the specimens *Oncorhynchus mykiss*, *Salmo trutta*, and *Perca fluviatilis* respectively. Correlation analysis between PFAS concentration and fish size in 121 *Salmo trutta* specimens revealed significant positive correlations for perfluorobutane sulfonic acid (PFBS), perfluorodecanoic acid (PFDA), and perfluorohexane sulfonic acid (PFHxS), and a negative correlation for perfluoropentanoic acid (PFPeA). These results underscore the critical need for continuous monitoring and regulatory efforts to mitigate PFAS exposure risks to both ecosystems and human health.

**Supplementary Information:**

The online version contains supplementary material available at 10.1007/s00216-024-05524-1.

## Introduction

Perfluoroalkyl and polyfluoroalkyl substances (PFAS) represent a class of anthropogenic chemicals widely used for their water-repellent and oleophobic properties in numerous industrial and consumer products. Because of their persistence in the environment and their ability to bioaccumulate, PFAS have become global contaminants, present in multiple environmental matrices, including freshwater sources [1]. Their alarming prevalence has raised significant questions about potential impacts on human health and ecosystem [2, 3]. In particular, PFAS exposure has been associated with different types of cancer, developmental toxicity, and immunotoxicity [4]. In freshwater ecosystems, fish are particularly vulnerable to PFAS exposure because of their location in trophic systems and their direct exposure to dissolved and particulate contaminants [5]. Therefore, analysis of PFAS in freshwater fish is critical to understanding bioaccumulation dynamics, ecotoxicological effects, and potential toxicological implications for fish fauna and human health through consumption of contaminated fish [3, 6]. In this context, many studies have highlighted the importance of fish as bioindicators of PFAS presence in the environment [7, 8]. Recent studies have shown how PFAS can adversely affect fish health by inducing endocrine, immunological, and reproductive alterations. This has been shown both in the laboratory on zebrafish and environmental studies [9–11]. These effects are largely related to the bioaccumulation of these compounds in lipid tissues, from where they act by interfering with fat metabolism and pancreatic activity [1, 10, 12].

The broad class of these compounds and the variability of biological responses necessitate a holistic approach to assess their environmental impact. This is especially important because fish are a source of PFAS assimilation in humans through consumption [13–15]. There are a number of studies reporting selective sampling and correlation between sampling location, human activities, pollution mock, and PFAS concentration [12, 13, 16–20].

In a recent study published by Jaus et al. in 2023 [21], 83 fish samples from lakes and streams in Switzerland were analyzed. The analysis showed the main presence of 5 PFAS (i.e., perfluorooctane sulfonate (PFOS), perfluorooctanoic acid (PFOA), perfluorononanoic acid (PFNA), perfluoroundecanoic acid (PFUnDa), and perfluorodecanoic acid (PFDA)) out of 15 PFAS analyzed with PFOS exceeding European Union (EU) limits in 4 samples. In general, other available data on the status of PFAS contamination of fish in Swiss reservoirs and watercourses are scarce and often refer to studies conducted on border water sources between Switzerland and France, Italy, or Austria [19, 21, 22].

The quantitative determination of PFAS in fish tissue requires effective and reliable extraction methods to ensure the accuracy and repeatability of analytical results. In addition, reaching low limits of quantification is often challenging because of the complexity of the matrix. Commonly used methods include solid phase extraction (SPE) and the QuEChERS (Quick, Easy, Cheap, Effective, Rugged, and Safe) extraction [5, 8, 16, 18, 19, 23–25]. The SPE method is widely used for pre-concentration and purification of PFAS from aqueous and biological samples, and is particularly effective in reducing the presence of interferents and improving the detection limits of analytes [9, 21, 24]. Initially developed for pesticide analysis, the QuEChERS method has also been adapted for the extraction of a wide variety of compounds including some PFAS. This method is based on two main steps, i.e., solvent extraction procedure (namely, salting out) and a purification step (namely, cleanup) using salts that reduce the miscibility and improve the separation between aqueous matrices and organic solvents and absorbing powders for dispersive solid phase extraction (dSPE), respectively. QuEChERS is appreciated for its simplicity, rapidity, and relatively low cost. In addition, thanks mainly to the cleanup phase, it is possible to reduce the matrix effect for complex matrices such as foodstuffs [8, 25–28]. QuEChERS preparation has the great advantage of being more flexible than SPE, allowing the analysis of a wide spectrum of compounds, ranging from drugs, to plant protection products, through precise PFAS [25, 28–30]. This makes the QuEChERS method even more sustainable than the SPE for carrying out various types of analysis without upsetting laboratory procedures or buying over specific, target-related solid phases as is the case for the SPE [16, 19, 31, 32]. In this study, a method of extracting fish flesh samples using QuEChERS was therefore optimized and employed for PFAS content assessment.

The determination of 15 PFAS contained in the fillet of more than 200 fish caught in Swiss lakes, ponds, and lowland rivers is reported in this study. In particular, the fish analyzed belong to the six most common fish species in Switzerland: *Coregonus wartmanni* (whitefish), *Cyprinus carpio* (common carp), *Oncorhynchus mykiss* (rainbow trout), *Perca fluviatilis* (perch), *Salmo trutta* (brown trout), and *Squalius cephalus* (common chub) [19, 21, 33, 34]. These fish species all have food interest and are subject to fishing. In this context, it is known that in food fisheries, the species plays a key role in the organoleptic quality of the catch, as does the size of the fish, which is proportional to the age of the animal and environmental conditions [35]. Several studies have explored the relationship between the size of the animal and the content of PFAS, with no consensus emerging [8, 12, 17, 36, 37]. A correlation between size and content of major PFAS was also carried out for fish of the species *Salmo trutta*, the most represented in this study and for which the size was available. The concentration of PFAS in the edible part of fish was compared with the requirements of Commission Regulation (EU) 2022/2388 in addition to the tolerable weekly intake (TWI) set by the European Food Safety Authority (EFSA) of 4.4 ng/kg body weight per week for the sum of perfluorooctane sulfonate (PFOS), perfluorooctanoic acid (PFOA), perfluorononanoic acid (PFNA), and perfluorohexane sulfonic acid (PFHxS) [26].

## Materials and methods

### Materials and reagents

Analytes labeled PFAS standards were purchased from Neochema (namely, perfluorobutanoic acid (PFBA), perfluoropentanoic acid (PFPeA), perfluorohexanoic acid (PFHxA), perfluoroheptanoic acid (PFHpA), perfluorooctanoic acid (PFOA), perfluorononanoic acid (PFNA), perfluorodecanoic acid (PFDA), perfluoroundecanoic acid (PFUnDA), perfluorobutane sulfonic acid (PFBS), perfluorohexane sulfonic acid (PFHxS), perfluorooctane sulfonic acid (PFOS), perfluorooctane sulfonamide (PFOSA), perfluoropentanesulfonic acid (PFPeS), perfluorotetradecanoic acid (PFTA), and perfluorododecanoic acid (PFDoDa), more information are given in Table S1 of Supplementary Information (SI)). Isotopically labelled standards (surrogate standards) were obtained from Wellington Laboratories (namely, sodium perfluoro-1-(13C8)-octanesulfonate (PFOS 13C8), perfluoro-n-(1,2-13C2)-hexanoic acid (MPFHxA), perfluoro-n-[1,2-13C2] octanoic acid (M8PFOA)) (Table S2). Liquid chromatography-mass spectrometry (LC–MS)-grade acetonitrile (≥ 99.9%, ACN) and methanol (MeOH; ≥ 99.9%) were obtained from Honeywell. A Sartorius Arium^®^ water purification system was used for ultrapure water. Evian^®^ water was obtained from local supermarket. Formic acid < 98% p.a. was obtained from CarlRoth. Ammonia hydroxide 25% and activated carbon < 5 mm were obtained from Sigma-Aldrich. Chromabond QuEChERS Mix XII composed of 4 g MgSO_4_ and 1 g NaCl (extraction, 5 g) and Chromabond QuEChERS Mix XX composed of 1.2 g MgSO_4_ and 0.4 g diamino (i.e., primary secondary amine (PSA)) (clean-up, AOAC 2007.01, 1.6 g) were obtained from Macherey Nagel. Filter (0.45 µm 13 mm) was obtained from BGB (Boeckten CH). Polypropylene tubes were purchased from CELLSTAR^®^ Greiner Bio-One.

### Fish samples

This study is based on fillet analysis of 218 fish specimens intended for human consumption, caught in Switzerland between 2022 and 2024. The fish were mostly donated by recreational fishermen, or came from fishing and monitoring campaigns organized by public entities. The following inclusion criteria were assessed: the fish were harvested following laboratory recommendations to avoid PFAS contamination, the fish come from areas where fishing is not restricted, the fish were caught in lowland lakes, ponds, and rivers (< 2000 m above sea), and come from areas with comparable degree of urbanization [19]. Therefore, the following exclusion criteria were observed: fish species of which too few individuals are available (< 5), fish that come from no-fishing zones due to PFAS pollution, fish that were taken or prepared without following laboratory instructions, fish caught at > 2000 m above sea. To minimize the risk of PFAS contamination, it was recommended to collect fish in special high-density polyethylene (HDPE) bags that have been previously analyzed for the absence of PFAS. Fishermen were instructed to eviscerate fish immediately after capture, carefully avoiding damage to the liver, stomach, and other systems. They were also asked to inspect the fish visually and discard any that were non-compliant, to prevent contamination of the fillet by bile acids such as cholic acid, digestive juices, or feces, which could lead to matrix effects or false positive results [38, 39]. Regarding the preparation of fish to separate the fillet for analysis from the waste parts, the laboratory either provided the necessary materials for fish preparation and tested free of PFAS (cutting board, knife, nitrile gloves, HDPE bags), or did the preparation in-house. The following fish species were analyzed: *Coregonus wartmanni* (*N* = 20), *Cyprinus carpio* (*N* = 11), *Oncorhynchus mykiss* (*N* = 11), *Perca fluviatilis* (*N* = 38), *Salmo trutta* (*N* = 131), and *Squalius cephalus* (*N* = 7). Thanks to the participation of authorities and amateur fishermen, it was also possible to know the size of 121 *Salmo trutta* out of 131. This information was used for the correlation study between size and PFAS content.

### Sample preparation

The fillet of each sample was collected and homogenized using a Satrap Coira blender. 10 ± 0.5 g of each sample was weighed and collected in a 50-mL polypropylene (PP) Falcon tube, then frozen at − 20 °C before extraction. PFAS concentration was expressed per mass of fillet, based on the mass of each sample.

### Extraction protocol

Samples were extracted using an optimized QuEChERS extraction method. For each sample, a PP Falcon tube containing the fillet was thawed and 7 mL of Evian^®^ water was added to each tube and the mixture was shaken. A volume of 70 µL of isotopically labeled standard solution at 0.1 mg/kg (surrogate standards) was added. Then, a volume of 10 mL of acetonitrile acidified with 150 µL of formic acid was added. The Falcon tube was vigorously mixed and vortexed. The XII Mix Chromabond^®^ QuEChERS was added and the falcon tube was shaken for 1 min. The mixture was centrifugated at 241 RCF for 10 min. A volume of 7 mL of the supernatant was collected and added to XX Mix Chromabond^®^ QuEChERS. The mixture was shaken and vortexed, then filtered with a 0.45 µm 13 mm filter in a PP Falcon tube. Solvent was removed in an oven at 60 °C for 12 h, and then the crude was redissolved in 1 mL of methanol:H_2_O (70:30) solution and added in the PP Falcon tube. The solution was filtered on 0.45 µm 13 mm filter in a 1.5-mL glass vial for LC analysis.

### Chemical analysis

Chemical analyses were carried out by liquid chromatography with tandem mass spectrometry (LC–MS/MS) on an LCMS-8060NX instrument (Shimadzu) with SB C-18 column (4.6 × 150 mm, 2.7 µm, Agilent). The column temperature was set at 40 °C. The volume injected was 20 µL. A binary gradient with a flow rate of 0.3 mL min^−1^ was used. Mobile phase A was made of 95% of water, 5% of MeOH, and 5 mM of ammonium acetate, while mobile phase B contained 5% of water and 95% of MeOH. The gradient was as follows: 0% of B at first, and increased to 100% by 5 min, a plateau up to 6 min, then from 100 to 0% in 2 min and a plateau up to 11 min. Mass spectrometric detection was carried out on a triple quadrupole LC–MS/MS system (8060 system Shimadzu Scientific, Inc., Columbia, MD, USA). The mass spectrometer was operating with electrospray ionization with negative polarization mode (ESI-). The multiple reaction monitoring (MRM) and compound-dependent parameters such as mass transition, collision energy (CE), and retention time are illustrated in Table S3.

### Quality assurance/quality control

A procedural blank including all solvents and equipment used for the sample preparation was run every extraction batch, and a duplicate and a spiked sample were included each 10 samples. To exclude loss and degradation of PFAS standards during the extraction procedure, spikes at 0.1 and 0.5 mg/kg were performed in 7 mL of Evian water and extracted according to the reported procedure. The values were compared with a direct curve in solvent. The recoveries repeated in triplicate are shown in Table S4 of Supplementary Information. A calibration curve subjected to the same extraction treatment as the samples is performed between 0.001 and 10 mg/kg range and showed slope ratios > 0.995. Samples with concentration of a target compound exceeding the highest point of the calibration curve were diluted and reanalyzed, for the final value expressed in mg/kg the dilution factor was considered. Quality assurance and quality control procedures included the use of appropriate internal standards in each sample and the addition of standards at a concentration of 0.1 or 0.5 mg/kg for every 10 samples analyzed to determinate method recoveries and ensure the accuracy of quantification. The limits of detection (LODs), calculated using the signal-to-noise ratio of 3:1, ranged from 0.001 to 0.02 mg/kg. The limits of quantification (LOQ) were determined according to sample recoveries at low concentrations [25] (0.007, 0.05, 0.1 μg/kg) that met the experimental criteria with recovery rates (R%) between 70 and 130%. The LOQs were found to range from 0.007 to 0.05 mg/kg.

### Statistical analysis and box-whisker plots

Shapiro–Wilk tests were conducted to check for normality within groups. An unpaired two‐sample *t* test, following an *F* test for variance homogeneity, was used to evaluate significant differences between two normal distributed and homogeneous sets of data. The correlations between size in centimeters and PFAS concentrations in fillet were assessed by Spearman correlation analysis. Significance was set at *α* = 0.05 in all tests. Box-whisker plots include median (horizontal line), mean (symbol “x”), median of the 3rd quartile, median of the 1st quartile, maximum and minimum values in the dataset, and outliers (symbol “•”). Quartile calculation is based on exclusive median.

## Results and discussion

### Extraction of PFAS from fish fillet

The choice of the most appropriate extraction method depends on several factors, including the type of sample, the concentration range of PFAS of interest, and the need to minimize the influence of the interferents [24, 26]. Method reliability is crucial not only to ensure data accuracy, but also to comply with regulatory standards, such as EFSA recommendations and European requirements, and to provide valid information for ecotoxicological and human health risk assessments [28, 40, 41]. A reliable method also allows monitoring the effectiveness of environmental management policies and regulation of PFAS use, thereby contributing to the protection of aquatic ecosystems and public health [3]. In this work, QuEChERS extraction was chosen for its ease of use but, more importantly, for its flexibility in terms of compounds that can be analyzed with this type of extraction [16, 21, 24, 26, 28]. The performance of the extraction method is evaluated on the basis of the recoveries rates. In fact, recovery rate assessment in doped samples is recognized as the best approach to evaluate the performance of an analytical method, particularly in the case of complex matrices [29, 42, 43]. The recoveries found were between 75 and 115% and were calculated as the arithmetic mean of all the recoveries analyzed. The detailed values for each target compound are reported in Table S5 of Supplementary Information.

### PFAS levels in fish

The dataset presents PFAS contamination in fish examined between 2022 and 2024. The results are shown in Table 1 as average concentration per species analyzed, corresponding standard deviation, and detection frequency in the population. In terms of diversity of PFAS, the species *Perca fluviatilis* has the highest diversity of compounds (positive for all PFAS tested), followed by *Salmo trutta*, *Oncorhynchus mykiss*, and *Cyprinus carpio* (9 PFAS out of 15) (Table 1). In particular, *Perca fluviatilis* is the only species of this study in which PFHpS, PFPeS, PFTA, PFOSA, and PFDoDA were detected. *Perca fluviatilis* is a species known to bioaccumulate a wide variety of different PFAS in comparison with other freshwater fish species [12, 15, 21, 22], as confirmed in this study. The variety and the high concentrations of PFAS in *Perca fluviatilis* are associated, according to the literature, at least in part with its purely carnivorous diet, that places this species in a trophic level particularly sensitive to this type of pollutants [12]. Also, within the framework of this study, *Perca fluviatilis* is the only species that has a purely carnivorous diet, feeding on zooplankton, macroinvertebrates, and fish species.

**Table 1:** Summary of concentrations of per- and polyfluoroalkyl substances in fish fillets. *Av*, average concentration in µg/kg; *SD*, standard deviation; *F* (%), frequency of positives (> LOQ); “-”, not quantified (< LOQ)

	*Coregonus wartmanni* (*N* = 20)		*Cyprinus carpio* (*N* = 11)		*Oncorhynchus mykiss* (*N* = 11)	
	Av (µg/kg) (min–max)	*F* (%)	Av (µg/kg) (min–max)	*F* (%)	Av (µg/kg) (min–max)	*F* (%)
PFBS	0.08 (0.04–0.13)	45%	-		-	
PFDA	0.74 (0.07–2.39)	95%	0.37 (0.09–1.52)	100%	0.02 (0.01–0.04)	64%
PFHpA	-		0.03 (0.02–0.04)	27%	0.02^a^	10%
PFHpS	-		-		-	
PFHxA	-		0.05 (0.02–0.07)	18%	0.03^a^	10%
PFHxS	5.07 (0.02–16.10)	60%	1.56 (0.06–14.63)	100%	0.44 (0.04–1.74)	80%
PFNA	0.16 (0.06–0.29)	50%	0.03 (0.02–0.03)	18%	0.07 (0.01–0.17)	20%
PFOA	-		0.02 (0.02)	9%	0.03 (0.02–0.03)	40%
PFOS	20.12 (0.69–109.90)	100%	3.58 (0.10–6.77)	64%	4.56 (0.13–19.40)	100%
PFOSA	-		-		-	
PFPeA	-		0.05 (0.04–0.05)	18%	0.50 (0.28–0.94)	100%
PFPeS	-		-		-	
PFTA	-		-		-	
PFDoDA	-		-		-	
PFUnA	0.32 (0.06–0.74)	40%	0.03 (0.01–0.05)	18%	0.02 (0.01–0.02)	70%
	*Perca fluviatilis* (*N* = 38)		*Salmo trutta* (*N* = 131)		*Squalius cephalus* (*N* = 7)	
	Av (µg/kg) (min–max)	*F* (%)	Av (µg/kg) (min–max)	*F* (%)	Av (µg/kg) (min–max)	*F* (%)
PFBS	0.05 (0.01–0.07)	11%	0.02 (0.01–0.04)	5%	0.01 (0.01–0.02)	29%
PFDA	1.43 (0.04–4.60)	79%	0.37 (0.01–3.18)	100%	0.50 (0.02–1.56)	57%
PFHpA	0.02 (0.010–0.10)	18%	0.03 (0.01–0.11)	27%	0.40^a^	14%
PFHpS	0.20 (0.10–0.30)	11%	-		-	
PFHxA	0.22 (0.01–1.10)	26%	0.05 (0.15–0.44)	18%	-	
PFHxS	0.46 (0.10–1.55)	47%	1.56 (0.01–11.10)	100%	0.60 (0.2–1.26)	71%
PFNA	0.18 (0.07–0.30)	39%	0.03 (0.01–0.27)	18%	0.10^a^	14%
PFOA	0.01 (0.01–0.02)	5%	0.02 (0.01–0.22)	9%	-	
PFOS	19.17 (0.01–41.50)	71%	3.58 (0.01–157.00)	64%	6.40 (1.16–21.30)	100%
PFOSA	0.10 (0.10–0.20)	5%	-		-	
PFPeA	0.28 (0.01–1.21)	26%	0.05 (0.01–3.54)	18%	0.10^a^	14%
PFPeS	0.16 (0.10–0.20)	5%	-		-	
PFTA	0.30 (0.20–0.40)	16%	-		-	
PFDoDA	0.45 (0.30–0.60)	16%	-		-	
PFUnA	0.54 (0.03–2.70)	63%	0.03 (0.01–0.59)	18%	0.40 (0.38–0.40)	29%

^a^The positive result refers to only one specimen

**Fig. 1:**
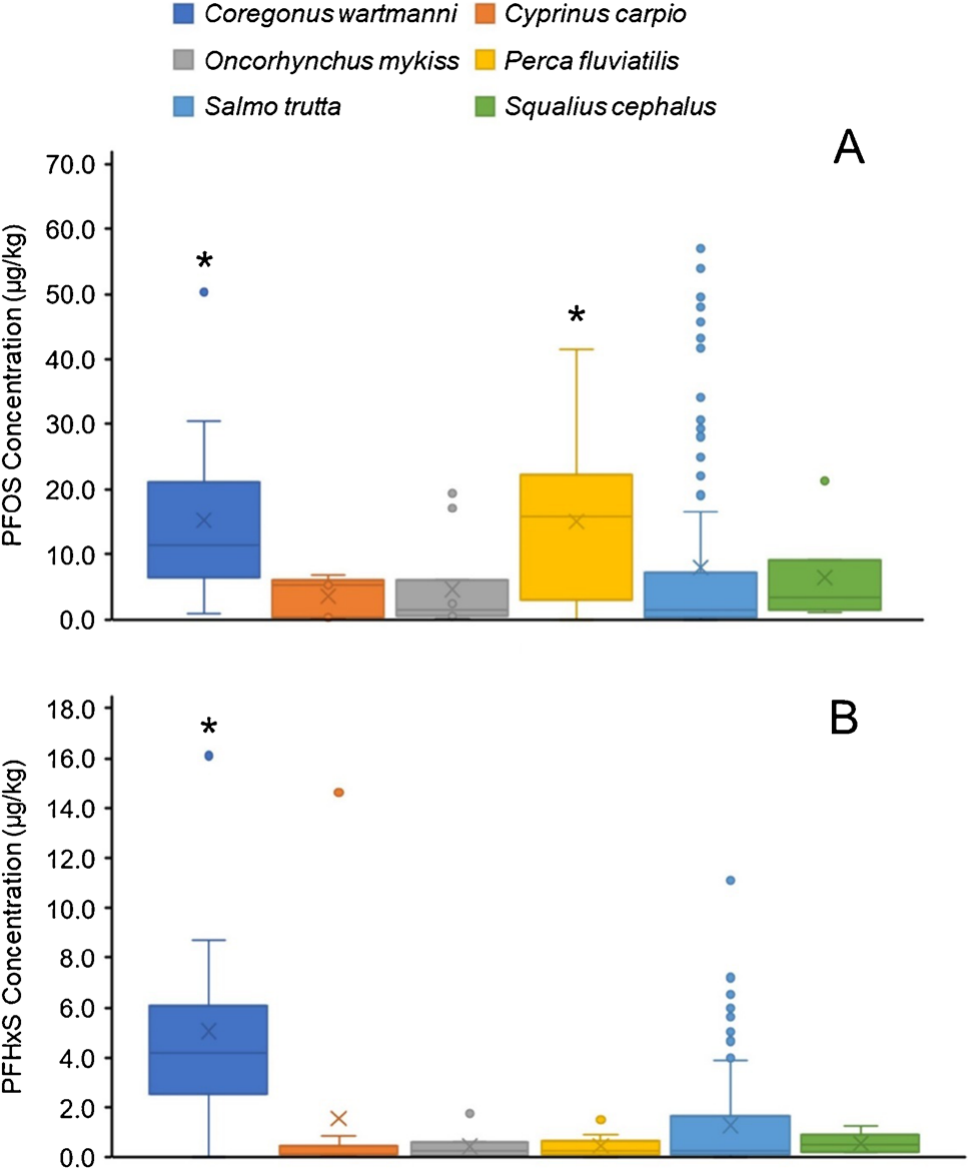
Box‐whisker plot of the fillet PFOS (**A**) and PFHxS (**B**) concentration. PFOS and PFHxS concentration is compared between species by a *t*-test (*p-value < 0.05). PFOS concentration is significantly higher in *Coregonus wartmanni* and *Perca fluviatilis* than in the other species (*p*-value < 0.05) but does not differ between these two species. PFHxS concentration is significantly higher in *Coregonus wartmanni* species (*p*-value < 0.05) in comparison to the other species

The PFAS present at the highest concentrations in all fish species examined is PFOS, followed by PFNA and PFHxS (Table 1). This is consistent with what has been described in the literature, particularly regarding the presence of PFOS at higher concentration than other PFAS [7, 12, 13, 17, 19–21]. The concentration of PFOS is significantly higher in the species *Coregonus wartmanni* and *Perca fluviatilis*, in comparison with the other species (*p* < 0.05) (Fig. 1A). The PFOS concentrations found in this study are comparable with what has been observed in studies conducted in the same geographical areas with values between 3.7–37.7 µg/kg and 2–20 µg/kg found by Jaus et al. and Valsecchi et al., respectively [19, 21]. *Coregonus wartmanni* species also shows the highest PFHxS values (*p* < 0.05) (Fig. 1B). In contrast, PFNA concentration is not significantly different among the species and its concentrations remain 10 to 100 times lower than PFOS, i.e., which is comparable with what has been described in the literature (Figure S1) [6, 19, 21]. None of the PFAS found at lower concentrations (below 5 µg/kg) shows significant differences between species. In terms of frequency, there are six most frequent PFAS found in the fish analyzed: PFOS, PFHxS, PFDA, PFPeA, PFUnA, and PFNA. PFOS is the most frequently detected in all species while the frequency of the other five compounds varies between species [5, 19–21].

In particular, a high detection frequency for PFHxS was observed in this study in contrast to the study of Valsecchi and colleagues, which was conducted in Switzerland and Italy but on different fish species. Valsecchi et al. showed a higher frequency of the compound PFDoDA, which was only found in *Perca fluviatilis* in this study. Differences in PFAS concentration can be associated both with differences in metabolism among the fish species considered, diet and plasma lipid and protein content, as well as with specificities related to anthropogenic pollution of fishing areas [5, 12, 18, 44, 45]. Regarding the species-specificity, recent studies show how the serum proteome and serum protein concentration contribute to the difference in PFAS bioaccumulation. This is related to the type of serum protein most present in fish, which is not always albumin and whose type can vary among species. Serum proteins are responsible for the transport of PFAS as well as fatty acids, so they directly affect their bioaccumulation [8, 37, 44]. Furthermore, it is known that PFDoDA is preferentially accumulated in the liver compared with the fillet. In the case of PFHxS, on the other hand, an increased frequency of detection in the fillet with respect to the liver is shown [12, 17, 20, 46]. The presence of PFAS in fish is also directly related to the type of PFAS found in the environment, the concentrations, and varieties of which can change greatly even within the same geographic areas [2, 3, 5, 17, 46].

### Correlation between PFAS concentration and fish size

The size of a fish is proportional to its age and developmental condition and to the quality and quantity of available nutrition [8, 16, 17, 20, 23, 36]. Size also determines the quality of the catch. Indeed, for the same species, the larger a specimen is, the greater its interest in terms of food [45]. In this study, the size data of 121 among the 131 specimens of *Salmo trutta* were available. This allowed a correlation between PFAS content and specimen size (Table 2). Correlation was made only for PFAS that were measured > LOQ in this species, namely PFBS, PFDA PFHpA, PFHxA, PFHxS, PFOS, and PFPeA. No correlation was observed between specimen size and concentration of PFOS, PFHpA, and PFHxA (Table 2). In contrast, the compounds PFBS, PFDA, and PFHxS showed a positive correlation between size and content (Table 2 and Figure S2). Interestingly, the compound PFPeA, on the other hand, shows a negative correlation between size and quantity (Table 2 and Figure S2).

**Table 2:** Correlation between fish size and PFAS concentration for 121 *Salmo trutta* specimens. Correlation is calculated using a Spearman correlation analysis. All data are integrated (where < LOQ was considered as = 0). *F* (%), frequency of positives; *r*, correlation coefficient; *nd*, not detected; *ns*, not significant (*p*-value > 0.05)

PFAS	*F* (%)	Correlation	Correlation type	*r*	*p*-value
PFBS	32%	Yes	Positive	0.23	0.013
PFDA	70%	Yes	Positive	0.61	< 0.001
PFHpA	16%	No	nd	0.05	ns
PFHxA	17%	No	nd	0.1	ns
PFHxS	74%	Yes	Positive	0.34	< 0.001
PFOS	100%	No	nd	0.09	ns
PFPeA	88%	Yes	Negative	− 0.47	< 0.001
∑PFAS	100%	No	nd	0.11	ns

The correlation between fish size and PFAS content is discussed in several studies without an unambiguous consensus emerging, except in the case of PFOS, where evidences suggested that there is no correlation between content and fish size, as confirmed by this study [8, 16, 17, 20, 36]. Based on the literature reviewed, this is the first time that a negative correlation has been shown between a PFAS, in this case PFPeA, and fish size. This statistically robust finding is likely to be associated with the fact that the compound PFPeA is a short-chain (C5) PFAS, and these kind of PFAS are known to poorly bioaccumulate and are characterized by a short half-life in animals [7, 9, 15, 18, 20, 21]. PFPeA is used to replace more toxic PFAS, along with PFBS, which, however, has bioaccumulation potential [11, 47]. The relationship between bioaccumulation potential and fish size with regard to some of the PFAS replacements is thus suggested by this study. In fact, PFBS shows a positive correlation, likely due to its bioaccumulation potential, as opposed to PFPeA. PFHxS content is also characterized by a positive correlation between fish size and content of this PFAS, which is consistent with the bioaccumulation characteristics described in the literature for this compound [5, 8, 15, 37].

### Food quality of fish

Fish are considered an extremely valuable food source in relation to their nutritional content, particularly due to their richness in protein, omega-3 fatty acids, vitamins (such as D and B12), and minerals (such as iodine, selenium, and zinc) [48–50]. Nevertheless, due to their position in the food chain, the nutritional quality of fish is often affected by the presence of bioaccumulated pollutant in the flesh [19, 34]. This also relates to PFAS pollution. Indeed, fish are known to bioaccumulate PFAS to the point that in some cases authorities have decreed no-fishing zones in highly polluted waters, with the aim of preserving the health of the population [7, 9, 12, 45]. For this reason, the European Commission has set limit values for PFOS, PFOA, PFNA, and PFHxS content in a number of fish [26] including the species *Coregonus wartmanni*, *Oncorhynchus mykiss*, *Perca fluviatilis*, and *Salmo trutta* (EU 2022/2388) [26]. The percentage of fish analyzed in this study whose concentration of PFOS, PFOA, PFNA, or PFHxS exceeds the requirements of EU 2022/2388 is shown in Table 3. None of the tested fish exceeded the requirements of EU 2022/2388 for PFOA and PFNA, while a greater percentage of samples analyzed exceeded the requirements for PFOS and PFHxS (Table 3). The relatively low concentration of PFOA and PFNA is likely attributable to the type of pollutants to which the fish are exposed, generally characterized by a higher prevalence of other PFAS, and the type of metabolism [1, 2, 11, 12, 21]. In fact, for all PFAS, bioaccumulation is greater in the liver than in muscle and this is particularly significant in the case of PFOA, which explains the low concentration of this compound in the fillet [8, 36, 37].

**Table 3:** Percentage of fish exceeding the requirements of EU regulation 2022/2388 (% Sample > EU Reg.) and maximum levels in μg/kg wet weight according to EU regulation 2022/2388 (EU Reg. (μg/kg)). Species *Cyprinus carpio* and *Squalius cephalus* are not listed in EU regulation 2022/2388 therefore not shown in the table

		*Coregonus wartmanni*	*Oncorhynchus mykiss*	*Perca fluviatilis*	*Salmo trutta*
PFOS	% Sample > EU Reg	5%	18%	5%	24%
	EU Reg. (μg/kg)	35.0	7.0	35.0	7.0
PFOA	% Sample > EU Reg	0%	0%	0%	0%
	EU Reg. (μg/kg)	8.0	1.0	8.0	1.0
PFNA	% Sample > EU Reg	0%	0%	0%	0%
	EU Reg. (μg/kg)	8.0	2.5	8.0	2.5
PFHxS	% Sample > EU Reg	50%	45%	5%	27%
	EU Reg. (μg/kg)	1.5	0.2	1.5	0.2
∑PFOS, PFOA, PFNA PFHxS	% Sample > EU Reg	10%	18%	0%	24%
	EU Reg. (μg/kg)	45.0	8.0	45.0	8.0

The compounds PFOS, PFOA, PFNA, and PFHxS are known to be particularly problematic in toxicological terms [2, 15, 35, 47]. In fact, these compounds have been associated with developmental disorders and adverse effects on serum cholesterol, liver, immune system, and birth weight [2, 3, 11]. For this reason, EFSA has set a tolerable weekly intake (TWI) of 4.4 ng/kg body weight per week for the sum of PFOS, PFOA, PFNA, and PFHxS [15, 26]. TWI is related to the maximum amount that can be ingested weekly without posing a significant risk to health. Considering an amount of 200 g of fish fillet for a person of 70 kg body weight, the TWI would be exceeded by a considerable percentage of the fish analyzed in this study, as shown in Table 4 [48]. In particular, TWI is exceeded in virtually all specimens of *Coregonus wartmanni* (95% of the specimens) and in all specimens of *Squalius cephalus* (100%), while it is exceeded in about one in two specimens for *Oncorhynchus mykiss*, *Perca fluviatilis*, and *Salmo trutta*. Exposure to PFAS, particularly PFOS, through the consumption of contaminated food that exceeds the TWI can lead to long-term health risks [6]. These risks are partly due to the bioaccumulation of PFAS in organs such as the liver and kidneys. PFAS mainly disrupt fat and carbohydrate metabolism, leading to increased serum cholesterol levels, which raise the risk of cardiovascular diseases. Additionally, they cause elevated serum alanine transaminase (ALT) levels and reduced birth weight [51–53]. While this information primarily relates to PFOS exposure, it likely applies to other PFAS as well, and further research is needed to confirm this [6, 53].

**Table 4:** Tolerable weekly intake (TWI) set by EFSA here calculated for a person of 70 kg body weight and an intake of 200 g of fish fillet

	*Coregonus wartmanni*	*Oncorhynchus mykiss*	*Perca fluviatilis*	*Salmo trutta*	*Cyprinus carpio*	*Squalius cephalus*
TWI (70 kg body weight, 200 g of fillet)	95%	55%	58%	50%	36%	100%

## Conclusions

The purpose of this work was to provide insight into the level of PFAS in fish from lowland water in Switzerland, focusing on the highest food quality part of the fish, the fillet. The fish tested represent a random sample of what might be food-caught fish. Samples were extracted using an optimized QuEChERS method that is robust and reliable for PFAS quantification even in complex matrices such as fish flesh, thus confirming its great potential. The data obtained were compared with European limits and requirements [26].

Overall, the data of this study highlight the significant bioaccumulation of PFAS compounds such as PFOS, PFOA, PFNA, and PFHxS in the analyzed fish species, confirming the bioaccumulation phenomenon observed in these animals [7, 9, 12].

Regular consumption of fish within a balanced diet is important for health in terms of providing long-chain n-3 fatty acids, as well as protein, peptides, vitamin D, selenium, phosphorus, and calcium [49, 50]. Adequate intake of these substances is possible with regular fish consumption, ideally as a minimum twice a week [16, 47, 50]. At this level of consumption, depending on the species of fish, toxicological concerns may emerge with TWI exceedances for PFAS. This study intended to contribute to a better description of the issue by highlighting how the choice of fish species can contribute to an excessive intake of PFAS through the diet. In fact, fish can accumulate PFAS to levels that can pose health risks [9, 15, 35]. This is particularly evident in fish species like *Coregonus wartmanni* and *Squalius cephalus*, where nearly all samples tested in this study exceeded the TWI established by the EFSA. This study also suggests that the use of low-bioaccumulative PFAS substitutes, such as PFPeA, is confirmed to be encouraging in terms of presence and accumulation in fish, and thus risk to human health. In fact, this compound not only has low concentrations in fish, but also a negative correlation between the size of the animal and the concentration in the edible portion. Further studies are needed to monitor the evolution of PFAS content in fish as a function of restrictions on the use of these compounds in industrial applications, as well as the use of replacement PFAS characterized by lower toxicity and bioaccumulation.

## Supplementary Information

Below is the link to the electronic supplementary material.

Supplementary file1 (DOCX 340 KB)
