# Supplementary material for: Determination of per- and polyfluoroalkyl substances (PFAS) in six different fish species from Swiss lakes
Source: Anal Bioanal Chem. 2024 Oct 1;416(28):6377–86. doi: 10.1007/s00216-024-05524-1 (PMC11541325; doi:10.1007/s00216-024-05524-1)
Supplement: Supplementary file 1 — Supplementary file1 (DOCX 340 KB) [file 216_2024_5524_MOESM1_ESM.docx]

***Supplementary Information***

**Determination of Per- and Polyfluoroalkyl substances (PFAS) in six different fish species from Swiss lakes**

Mylène Soudani^1^, Lucie Hegg^2^, Camille Rime^3^, Camille Coquoz^4^, Denise Bussien Grosjean^5^, Francesco Danza^6^, Nicola Solcà^6^, Fiorella Lucarini*^1,7^ and Davide Staedler*^1,8^

^1^TIBIO Suisse romande, Chemin de Bérée 4C, 1010 Lausanne, Switzerland

^2^Center for Primary Care and Public Health (Unisanté), University of Lausanne, Route de la Corniche 2, 1066 Epalinges-Lausanne, Switzerland

^3^TIBIOLab Sàrl, Route d’Yverdon 34, 1373 Chavornay, Switzerland

^4^Département de la mobilité, du territoire et de l’environnement (DMTE), [Service de l’environnement (SEN)](https://www.vs.ch/fr/web/sen), Avenue de la Gare 25, 1950 Sion, Switzerland.

^5^Direction de l’environnement industriel, urbain et rural, Direction générale de l’environnement, Etat de Vaud, Chemin des Boveresses 155, 1066 Epalinges, Switzerland

^6^Dipartimento del Territorio, Divisione dell’Ambiente, Sezione della protezione dell'aria dell'acqua e del suolo, Via Franco Zorzi 13, 6501 Bellinzona, Switzerland

^7^School of Engineering and Architecture of Fribourg, Institute of Chemical Technology, HES-SO University of Applied Sciences and Arts of Western Switzerland, Boulevard de Pérolles 80, 1700 Fribourg, Switzerland

^8^Department of Biomedical Sciences, University of Lausanne, Rue du Bugnon 27, 1011 Lausanne, Switzerland

*corresponding authors: [fiorella.lucarini@hefr.ch](mailto:fiorella.lucarini@hefr.ch), [davide.staedler@unil.ch](mailto:davide.staedler@unil.ch)

**Table S1.** Standards employed for the calibration curve and sample spiking.

| **Compounds** | **Abbrev.** | **N° CAS** | **Concentration**  **µg/mL** | **Chemical purity %** |
| --- | --- | --- | --- | --- |
| Perfluorobutanoic acid | PFBA | 375-22-4 | 100 ±3.5 | 98.4 |
| Perfluoropentanoic acid | PFPeA | 2706-90-3 | 100 ±3.5 | 99.6 |
| Perfluorohexanoic acid | PFHxA | 307-24-4 | 100 ±3.5 | 99.0 |
| Perfluoroheptanoic acid | PFHpA | 375-85-9 | 100 ±3.5 | 99.0 |
| Perfluorooctanoic acid | PFOA | 335-67-1 | 100 ±3.5 | 99.0 |
| Perfluorononanoic acid | PFNA | 375-95-1 | 100 ±3.5 | 98.0 |
| Perfluorohexane sulfonic acid | PFHxS | 3871-99-6 | 100 ±3.5 | 98.5 |
| Perfluorobutane sulfonic acid | PFBS | 29420-49-3 | 100 ±3.5 | 99.9 |
| Perfluorooctane sulfonic acid | PFOS | 2795-39-3 | 100 ±3.5 | 99.0 |
| Perfluorodecanoic acid | PFDA | 335-76-2 | 100 ±3.5 | 99.1 |
| Perfluoroundecanoic acid | PFUnA | 2058-94-8 | 100 ±3.5 | 96.0 |
| Perfluorododecanoic acid | PFDoDA | 307-55-1 | 100 ±3.5 | 97.0 |
| Perfluoropentane sulfonic acid | PFPeS | 630402-22-1 | 100 ±3.5 | 95.8 |
| Perfluorooctane sulfonamide | PFOSA | 754-91-6 | 100 ±3.5 | 95.0 |
| Perfluorotetradecanoic acid | PFTA | 376-06-7 | 100 ±3.5 | 99.9 |

**Table S2.** Internal standards.

| **Compound** | **Abbreviation** | **Conc. µg/mL** | **Chemical purity %** | **Isotopical purity %** |
| --- | --- | --- | --- | --- |
| Sodium perfluoro-1-(13C8)-octanesulfonate | M8PFOS | 50 | <98 | <99 |
| Perfluoro-n-(1,2-13C2)-hexanoic acid | MPFHxA | 50 | <98 | <99 |
| Perfluoro-n-(13C8)- octanoic acid | M8PFOA | 50 | <98 | <99 |

**Table S3.** Details of MS analyses. Parameters for MRM detection in negative mode were: gas flow 15 L min^-1^; gas temperature 140°C; nebulizer gas 40 psi; sheath gas temperature 375°C; sheath gas flow: 12 L min^-1^; voltage of the MS capillary 2500 V in negative polarity.

| **Compounds** | **Target transition (Tg)**  **Precursor ion (m/z)** | **Production ion**  **(m/z)** | **Collision energy (V)** | **Qualifier transition**  **Precursor ion**  **(m/z)** | **Production ion**  **(m/z)** | **Retention time** |
| --- | --- | --- | --- | --- | --- | --- |
| Sodium perfluoro-1-(13C8)-octanesulfonate | 507.00 | 506.90 | 40 | 507.00 | 80.00 | 10.691 |
| Perfluoro-n-(1,2-13C2)-hexanoic acid | 315.00 | 119.00 | 11 | 315.00 | 223.00 | 7.723 |
| Perfluoro-n-(13C8)- octanoic acid | 421.10 | 375.95 | 10 | 421.00 | 172.05 | 9.856 |
| Perfluorobutanoic acid | 213.10 | 169.05 | 9 | 213.10 | 169.05 | 3.169 |
| Perfluoropentanoic acid | 263.00 | 219.05 | 8 | 263.00 | 69.00 | 6.400 |
| Perfluorohexanoic acid | 313.10 | 269.00 | 9 | 313.10 | 119.00 | 7.733 |
| Perfluoroheptanoic acid | 363.10 | 319.05 | 10 | 363.10 | 169.00 | 8.889 |
| Perfluorooctanoic acid | 413.00 | 369.95 | 10 | 413.00 | 169.05 | 9.767 |
| Perfluorononanoic acid | 463.00 | 418.95 | 10 | 463.00 | 219.00 | 10.660 |
| Perfluorohexane sulfonic acid | 399.00 | 98.95 | 47 | 399.00 | 79.95 | 8.919 |
| Perfluorobutane sulfonic acid | 298.90 | 79.90 | 34 | 298.90 | 98.95 | 6.765 |
| Perfluorooctane sulfonic acid | 498.90 | 79.95 | 59 | 498.90 | 98.90 | 10.676 |
| Perfluorodecanoic acid | 513.00 | 468.95 | 11 | 512.90 | 219.00 | 11.408 |
| Perfluoroundecanoic acid | 563.00 | 518.95 | 11 | 563.10 | 269.00 | 12.081 |
| Perfluorododecanoic acid | 612.90 | 569.00 | 11 | 612.90 | 169.00 | 12.638 |
| Perfluoropentane sulfonic acid | 349.00 | 79.90 | 10 | 349.00 | 98.90 | 7.918 |
| Perfluorooctane sulfonamide | 498.10 | 77.95 | 35 | 498.10 | 219.00 | 12.125 |
| Perfluorotetradecanoic acid | 712.90 | 669.10 | 10 | 712.90 | 169.00 | 13.574 |

**Table S4.** Mean recovery of spiked standard in water at 0.1 and 0.5 μg/kg in triplicate calculated with respect to calibration curve in solvent.

|  | **Recovery % (RSD, %)** |
| --- | --- |
| PFBS | 91% (3.5) |
| PFDA | 103% (5.4) |
| PFHpA | 97% (2.1) |
| PFHpS | 95% (4.3) |
| PFHxA | 90% (1.5) |
| PFHxS | 98% (6.3) |
| PFNA | 94% (4.1) |
| PFOA | 89% (3.9) |
| PFOS | 105% (6.3) |
| PFOSA | 87% (5.2) |
| PFPeA | 93% (4.6) |
| PFPeS | 111% (6.7) |
| PFTA | 85% (7.4) |
| PFDoDA | 96% (3.7) |
| PFUnA | 108% (5.8) |

**Table S5**. Mean recovery of PFAS calculated on 22 spiked samples at PFAS concentrations of 0.1 and 0.5 μg/kg.

|  | **QuEChERS extraction** |
| --- | --- |
|  | **Recovery % (RSD, %)** |
| PFBS | 110% (10.5) |
| PFDA | 115% (9.2) |
| PFHpA | 98% (8.5) |
| PFHpS | 91% (7.3) |
| PFHxA | 83% (2.8) |
| PFHxS | 106% (7.5) |
| PFNA | 96% (1.2) |
| PFOA | 98% (4.2) |
| PFOS | 98% (5.4) |
| PFOSA | 75% (6.9) |
| PFPeA | 90% (3.0) |
| PFPeS | 97% (4.3) |
| PFTA | 101% (8.8) |
| PFDoDA | 103% (6.7) |
| PFUnA | 102% (6.9) |


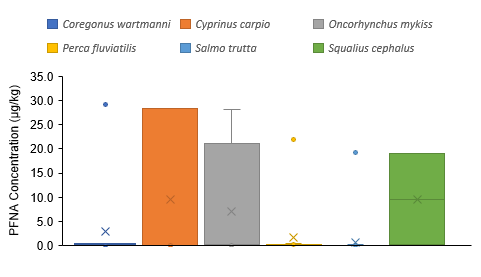


**Fig. S1.** Box‐whisker plot of the fillet perfluorononanoic acid (PFNA) concentration. Concentration is compared between species by a t-test: p-value >0.05 for all comparisons.

**Fig. S2.** PFAS and fish size correlation for PFDA (significant positive correlation), PFBS (significant positive correlation), PFHxS (significant positive correlation) and PfPeA (significant negative correlation). In red: trend line (linear).
